# Macrophage and IL-6 signaling modulate multiple myeloma progression and response to metformin and CAR-T cell therapy

**DOI:** 10.3389/fimmu.2026.1760136

**Published:** 2026-04-16

**Authors:** Xuanyou Liu, Iris Kemler, David Dingli

**Affiliations:** 1 Department of Molecular Medicine, Mayo Clinic, Rochester, MN, United States; 2 Division of Hematology, Mayo Clinic, Rochester, MN, United States

**Keywords:** CAR-T, CD126, IL-6, macrophages, metformin, multiple myeloma

## Abstract

The tumor microenvironment (TME) plays a crucial role in tumor progression and therapeutic response. Monocytes and macrophages, depending on their phenotype, can either enhance anti-tumor immunity or promote tumor growth by regulating cytokines such as IL-6. Multiple myeloma (MM), a hematologic malignancy, expresses the IL-6 receptor CD126 on its surface. Chimeric antigen receptor (CAR)-T cells targeting CD126 have been developed and shown to effectively eliminate CD126-positive tumor cells. However, interactions between macrophages, myeloma cells, and CAR-T cells require further investigation to optimize treatment strategies and minimize adverse effects. In this study, THP-1-derived macrophages, CD126 CAR-T cells, and MM cells, especially RPMI 8226 cells, were used to explore these interactions. A reduced cell population and an increased apoptosis of RPMI 8226 cells were observed in the presence of M1 macrophages. Metformin, known to influence IL-6 production, reduced the viability of RPMI 8226 cells with decreased CD126 expression. However, the killing activity of CD126 CAR-T cells against RPMI 8226 cells decreased after co-culture with macrophages, likely due to interactions between the CAR-T cells and the macrophages. These findings highlight the significant role of macrophages and inflammatory responses in MM progression and in modulating the efficacy of CAR-T cell therapy, providing valuable insights for the optimization of immunotherapeutic strategies.

## 1 Introduction

Multiple myeloma (MM) is a hematologic malignancy marked by the abnormal growth of plasma cells. Despite advancements in treatment, MM remains an incurable disease due to its complex biology and interactions within the tumor microenvironment (TME) (1). The TME, consisting of various immune cells, stromal cells, cytokines, and extracellular matrix components, plays a critical role in disease progression, immune evasion, and resistance to therapy (2). In the TME, macrophages are highly plastic and can adopt distinct phenotypic states based on signals from their surroundings (3). Tumor-associated macrophages (TAMs) are generally classified into two major phenotypes: M1 pro-inflammatory macrophages and M2 anti-inflammatory macrophages (3).

M1 macrophages play a crucial anti-tumor role in tumor progression, as they can eliminate tumor cells through phagocytosis and antibody-dependent cell-mediated cytotoxicity (3, 4). However, M2 macrophages typically exhibit an immune-suppressive profile, promoting tissue repair and facilitating tumor growth. Currently, M2-polarized macrophages have been linked to poor prognosis in several types of cancer, including epithelial ovarian cancer, pancreatic cancer, lung cancer, and MM (5–8). It is worth emphasizing that high CD163^+^ M2 macrophage density in the bone marrow of multiple myeloma patients, either at the time of initial diagnosis or relapse, represents an independent adverse prognostic marker and correlates with poor overall survival (9, 10). In contrast, increased infiltration of iNOS^+^ M1 macrophages correlates with better overall survival (9, 10). Augmenting M1 macrophage activity or reprogramming M2 macrophages to the M1 phenotype offers a promising approach to enhancing anti-tumor immunity and overcoming therapy resistance.

Chimeric antigen receptor T-cell (CAR-T) therapy has shown promising results in the treatment of various hematologic malignancies, such as MM, B-cell non-Hodgkin lymphoma, and leukemia (11, 12). CARs are composed of three fundamental domains: intracellular, transmembrane, and extracellular regions (13). Briefly, the transmembrane domain links extracellular and intracellular domains. The extracellular domain, usually composed of a single-chain variable fragment (scFv) enables CAR-T cells to bind selectively to target cells, while the intracellular domain facilitates T-cell activation. Although CAR-T cell therapy has demonstrated significant efficacy in treating hematologic malignancies, its long-term success is hindered by several challenges, including T-cell exhaustion and the suppressive effects of the TME (14). Through the production of immunosuppressive cytokines, depletion of key enzymes, and recruitment of regulatory T cells (Tregs), macrophages can inhibit T-cell-mediated anti-tumor responses (14, 15). Therefore, overcoming the immunosuppressive influence of macrophages provides a promising strategy to increase the durability and efficacy of CAR-T therapy in MM.

Among pro-inflammatory cytokines, interleukin-6 (IL-6) plays a key role in linking chronic inflammation to cancer by promoting tumor initiation, growth, and metastasis (16–18). Elevated IL-6 levels in the TME are associated with chronic inflammation, immune suppression, and poor prognosis in various cancers, including MM, breast, lung, and colorectal cancers (19–23). CD126, also known as IL-6 receptor alpha (IL-6Rα), is a membrane-bound receptor that mediates IL-6 signaling by forming a complex with glycoprotein 130 (gp130), leading to downstream activation of signal transducer and activator of transcription 3 (STAT3) and other tumor-promoting pathways (24, 25). Targeting the IL-6/CD126 pathway with monoclonal antibodies, such as Tocilizumab (an IL-6 receptor inhibitor), or CAR-T cells has emerged as a potential therapeutic strategy to block tumor-promoting inflammation and improve cancer treatment outcomes (26).

Metformin, a commonly prescribed medication for managing diabetes, has gained attention for its potential role in modulating immune responses in cancer. Studies have shown that metformin can alter macrophage polarization, shifting them from the tumor-promoting M2 phenotype to the anti-tumoral M1 phenotype (27). Meanwhile, metformin has been shown to modulate tumor-infiltrated immune cells, including both CD8^+^ and CD4^+^ T cells, as well as natural killer (NK) cells, in various tumor models (28). In a CAR-T cell therapy model, metformin promoted CAR-T cell cytotoxicity effects, leading to decreased tumor growth (29). The dual effect of metformin on both macrophages and T cells highlights its potential as an adjuvant therapy in MM treatment.

Considering the significant interaction between macrophages, CAR-T therapy, and metformin in tumor progression, along with the critical influence of IL-6 signaling and the elevated expression of CD126 on MM cells (30), the present study aims to investigate: 1) the role of IL-6 in MM cell proliferation and macrophage differentiation; 2) the effects of M1 and M2 macrophages on the growth of MM cells; 3) the potential impact of metformin on macrophage function and its association with MM cell proliferation; and 4) the interaction between macrophages and CD126 CAR-T cells in killing MM cells. To achieve these goals, MM cell lines, especially RPMI 8226 cells, were used in this study. Additionally, macrophages were differentiated from human monocyte THP-1 cells or human peripheral blood mononuclear cells (PBMCs), and CAR-T cells targeting the IL-6 receptor were generated for analysis.

## 2 Materials and methods

### 2.1 Cell culture

Human monocyte cell line THP-1, human multiple myeloma cell lines (RPMI 8226, MM1.S, and U266), and chronic myeloid leukemia cell line (K562) were either obtained from the American Type Culture Collection (ATCC) or Mayo Clinic Laboratories. All cell lines were maintained in RPMI-1640 medium (Thermo Fisher) at 37 ˚C under 5% CO_2_. The culture medium was supplemented with 10% fetal bovine serum (Thermo Fisher) and further enriched with penicillin (100 U/ml) and streptomycin (100 μg/ml). A third-generation CD126-targeted CAR was developed in our lab, containing the single-chain variable fragment (scFv) rhMP-1 (30). This fragment recognizes the same epitope as tocilizumab and competitively inhibits the binding of IL-6 to its receptor, IL-6R. Anti-human IL-6 was purchased from Invivogen (Cat #mabg-hil6-3).

### 2.2 Macrophage differentiation assay

THP-1 cells were differentiated with phorbol 12-myristate 13-acetate (PMA, Sigma-Aldrich, Cat #P8139). THP-1 cells were seeded in 96-well plates at varying densities of 1×10^4^, 5×10^4^, and 1×10^5^ cells/well, and treated with different concentrations of PMA (0, 25, 50, 100, and 150 ng/ml), followed by overnight incubation at 37 °C (**Supplementary Figures S1A, B**). Each experimental condition was performed in triplicate. In order to minimize the cytotoxicity of PMA and lipopolysaccharide (LPS), 50 ng/ml PMA and 10 ng/ml LPS were chosen for the following experiments. The next day, the cells were washed with phosphate-buffered saline (PBS) and cultured in fresh media for an additional 24 hours before exposure to polarizing cytokines. Macrophages were polarized to the M1 phenotype by incubating them with 20 ng/ml of Interferon-gamma (IFN-γ, PeproTech, Cat #300-02) and 10 ng/ml of LPS (Sigma-Aldrich, Cat #L4391) for 24 hours (31, 32). M2 polarization was induced by incubating macrophages with 20 ng/ml of IL-4 (PeproTech, Cat #200-04) for 48 hours, either alone or in combination with 20 ng/ml of IL-13 (PeproTech, Cat #200-13). THP-1 monocyte differentiation was initiated on separate days to obtain polarized M0, M1, and M2 macrophages on the same day. 6 well plate transwell inserts (membrane pore size of 0.4 μm, Corning, Cat #3450) were used for indirect co-culture experiments. 8×10^5^ THP-1 monocytes were differentiated on the inserts, which allowed the exchange of soluble factors but not the cells.

### 2.3 Cell proliferation and CAR-T cell cytotoxicity assay

Tumor cells and differentiated macrophages were co-cultured in RPMI-1640 medium at a 1:1 ratio for 24, 48 and 72 hours. Luciferase-expressing cancer cell lines were used as described previously (30). Bioluminescence was quantified at specific time points using a luminometer (Infinite M200 Pro, Tecan) to assess cell proliferation and viability, with results reported in relative light units. In parallel, the cell counting kit-8 (CCK8, Abcam, Cat #ab228554) was used to assess the proliferation and viability of cells lacking luciferase expression, according to the manufacturer’s instructions. To assess CAR-T cell–mediated killing activity, differentiated macrophages (M0, M1, and M2) were seeded in 96-well plates and co-cultured with RPMI 8226-Luc cells at a 1:1 ratio for 24 hours. Subsequently, CAR-T cells or mock untransduced (UTD) T cells were added at a 1:1 or 1:2 effector-to-target (E:T) ratio and incubated for an additional 24 hours. After co-culture, CAR-T cell cytotoxic activity against tumor cells was evaluated based on the remaining viable luciferase-expressing tumor cells. Wells containing tumor cells without T cells served as controls.

### 2.4 Quantitative real-time PCR

Total ribonucleic acid (RNA) was isolated from samples using the RNeasy Mini Kit (Qiagen, Cat #74104). Gene expression was analyzed by qRT-PCR using the LightCycler^®^ 480 RNA master hydrolysis probes (Roche, Cat #04991885001) on a LightCycler^®^ 480 PCR machine (Roche), following the manufacturer’s protocol. The mRNA expression was calculated using the comparative cycle threshold (2−ΔΔCt) method and reported as the relative fold change compared to the control, normalized with house-keeping genes (GAPDH). The primers for qRT-PCR are:

INOS

Forward: GCTCTACACCTCCAATGTGACC, Reverse: CTGCCGAGATTTGAGCCTCATG,

Probe:/56-FAM/TC ACA GCC T/ZEN/T TGG ACC TCA GCA AA/3IABkFQ/

CD163

Forward: TTTGTCAACTTGAGTCCCTTCAC, Reverse: TCCCGCTACACTTGTTTTCAC

Probe:/56-FAM/AG ACA AGG A/ZEN/G CTG AGG CTA GTG GA/3IABkFQ/

GAPDH

Forward: ACA ACT TTG GTA TCG TGG AAG G, Reverse: GCC ATC ACG CCA CAG TTT C

Probe:/56-FAM/AT CAC TGC C/ZEN/A CCC AGA AGA CTG TG/3IABkFQ/

### 2.5 Flow cytometry analysis

According to the manufacturer’s recommendations, Fc receptor blocking solution (Human TruStain FcX™, Biolegend, Cat #422301) was used to block Fc receptor–mediated nonspecific staining. After blocking, corresponding antibodies were added to each tube containing 1×10^5^ cells with a total volume of 100ul and incubated for 30 minutes on ice. Fixation/permeabilization buffer was used to permeabilize the cell membrane for intracellular staining according to the manufacturer’s recommendations (BD, Cat #554714). Briefly, cell pellets were resuspended in 100 µL PBS containing 2% FBS. Then, 300 µL of Fix/Perm solution was added and mixed, followed by incubation for 15 minutes at room temperature. Next, 1 mL of 1×Wash solution was added. Cells were centrifuged at 300 × g for 5 minutes, and the supernatant was discarded. The cell pellets were then resuspended, stained with the optimal concentration of intracellular antibodies, and incubated on ice for 30 minutes. A volume of 500 µL of PBS was added to each tube, followed by centrifugation at 300 × g for 10 minutes. The supernatant was carefully aspirated, and the cell pellet was resuspended in 500 µL of PBS. The resulting suspension was then transferred to flow cytometry tubes for subsequent analysis. PE or APC anti-human CD126 antibody (Biolegend, Cat #352803 or 352805) was used to detect CD126 expression on the MM cells (**Supplementary Figure S2A**). CD3 antibody (Biolegend, Cat #300415) was used to distinguish T cells from other CD3 negative cells (**Supplementary Figure S2B**). Monocyte-macrophage populations were identified by gating on CD11C-positive cells (BioLegend, Cat #301603 or 301605, **Supplementary Figure S2C**). T-cell activation and exhaustion markers CD25, Granzyme B, and programmed cell death protein 1 (PD-1) were purchased from BioLegend (Cat #302606, 372203, and 329920). Data acquisition was performed on an LSR Fortessa X-20 flow cytometer (BD Biosciences), and subsequent analysis was conducted using FlowJo v10 software (FlowJo LLC, USA).

### 2.6 Cell apoptosis analysis

Apoptosis of macrophages and MM cells was determined using an APC Annexin V and propidium iodide (PI) staining kit from Biolegend (Cat #640932) according to the manufacturer’s protocol. RPMI 8226 cells were co-cultured with macrophages for 24 hours. RPMI 8226 were gated from CD11C negative cells. Macrophages were co-cultured with CAR-T cells for 24 hours. Macrophages were gated from CD3 negative cells. Late apoptotic cells were defined as Annexin V and PI double-positive cells as described (33).

### 2.7 Measurement of inflammatory cytokines

Cell-conditioned supernatant was harvested at specific time points, and enzyme-linked immunosorbent assay (ELISA) was performed according to the manufacturer’s recommendation. The kits for C-X-C motif chemokine ligand 10 (CXCL10, Cat #439904), pro-inflammatory cytokines IL-6 (Cat #430504), and anti-inflammatory cytokines IL-10 (Cat #430604) were purchased from Biolegend.

### 2.8 Membrane tracking assay

Target cells, such as K562 cells, were first labeled with membrane dye BioTracker 555 (Sigma #SCT107) according to the manufacturer’s instructions. Labeled K562 cells were co-cultured with unstained cells, such as macrophages, at a ratio of 1:1, for 24 hours. Flow cytometry was conducted later to detect the BioTracker 555 signal with appropriate cell differentiation antibodies.

### 2.9 Metformin study

Metformin was purchased from Sigma (Cat #317240). RPMI 8226 cells were seeded in 12-well plates for flow cytometry and in 96-well plates for cell viability analysis at the end of macrophage polarization. Concurrently, varying concentrations of metformin (0, 1 and 5mM) were administered, with a total incubation period of 72 hours. To evaluate the effects of metformin on CAR-T cell activity against tumor cells, RPMI 8226 cells were pre-treated with metformin (1 or 5 mM) for 24 hours, followed by the addition of CD126 CAR-T or UTD T cells at E:T ratios of 1:1 or 1:2 for an additional 24 hours. In parallel, RPMI 8226 cells were treated with metformin and CAR-T cells simultaneously for 24 hours.

### 2.10 Phagocytosis study

Tumor cells (1×10^6^) were labeled with carboxyfluorescein succinimidyl ester (CFSE) at a final concentration of 5 µM (Invitrogen, Cat #C34570) for 20 minutes at room temperature or 37 °C, protected from light (34). Cells were washed and then co-cultured with THP-1-derived macrophages at a 1:1 ratio for 4 or 24 hours. The phagocytosis inhibitor cytochalasin D (final concentration 5 µM; Stemcell, Cat #100-0556) was applied to selected wells containing THP-1-derived macrophages 10 minutes prior to the addition of tumor cells. Tumor cells and macrophages were distinguished by CD11C expression, and phagocytosis efficiency (CD11C^+^/CFSE^+^ cells) was assessed by flow cytometry.

### 2.11 PBMC differentiation to macrophages

PBMCs were isolated from healthy donor leukocyte cones from the Mayo Clinic Blood Transfusion Center (Rochester, MN). Isolated PBMCs were plated at a concentration between 1-2×10^7^ cells/ml in RPMI-1640 medium and differentiated into macrophages as previously described (35). Briefly, the cells were incubated for 2 hours before non-adherent cells were removed through repeated washes in PBS. The cells were then incubated in complete RPMI-1640 medium with 100 ng/ml macrophage colony-stimulating factor (M-CSF, PeproTech, Cat #300-25) for 7 days. PBMCs were also used for the isolation of CD14-positive cells prior to differentiation using the human pan monocyte isolation kit (Biolegend, Cat #480060) according to the manufacturer’s instructions. The resulting monocytes were cultured at 1×10^6^ cells/ml in complete RPMI-1640 medium with M-CSF for 7 days. Positive CD14 expression and morphological changes from monocytes to macrophages were observed (**Supplementary Figures S3A, B**). On day 7, the macrophages were polarized with 20 ng/ml IFN-γ and 10 ng/ml LPS for M1, or 20 ng/ml IL-4 and IL-13 for M2, as previously described (35).

### 2.12 Statistical analysis

All the values were presented as the mean ± standard error of the mean (SEM). The experiments were repeated 3 times. The unpaired student’s t-test (two-sided) was used to compare data between two groups. One way ANOVA (analysis of variance) with Tukey’s *post hoc* analysis was used to compare data among three or more groups. Statistical analysis was performed using GraphPad Prism 4.0. The differences were considered statistically significant when a p value < 0.05.

## 3 Results

### 3.1 IL-6 stimulated the proliferation of RPMI 8226 cells

**Figure 1:**
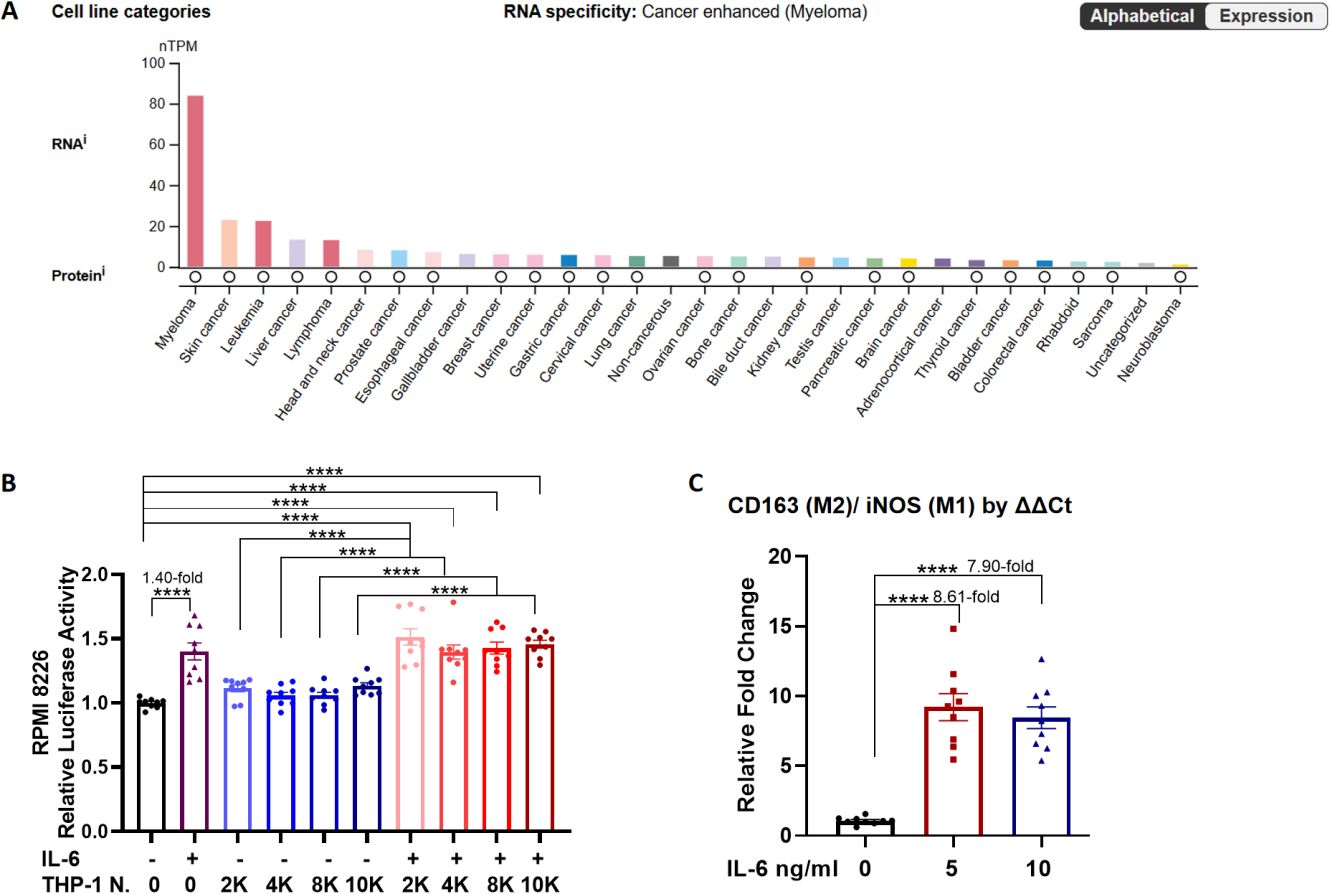
Effects of IL-6 on cellular proliferation. **(A)** RNA and protein expression of IL-6 receptor in various cancer cells from the HPA database (https://www.proteinatlas.org/ENSG00000160712-IL6R/cell+line, accessed on 29 Nov 2025). **(B)** Luciferase reporter assay for measuring RPMI 8226 cells activity with or without THP-1 cells, and IL-6 (5 ng/ml) for 48 hours. The seeding number of MM cells was 10,000. THP-1 cell numbers (THP-1 N.) were varied at 2000 (2K), 4000 (4K), 8000 (8K), and 10,000 (10K). **(C)** THP-1 cells were stimulated with IL-6 at concentrations of 0, 5, or 10 ng/ml for 24 hours. At the end of incubation, mRNA expression levels of iNOS and CD163 in THP-1 cells were quantified by qRT-PCR, and the ratio was determined by comparing the 2-ΔΔCt values of CD163 and iNOS. N = 9. ****p <0.0001.

Inflammation has been demonstrated to play a pivotal role in promoting cancer proliferation and differentiation, with IL-6 serving as a central mediator (16). In multiple myeloma, elevated serum IL-6 levels are closely associated with disease pathogenesis (36). According to the human protein atlas (HPA) database, among various cancer types, the IL-6 receptor mRNA expression is highest in myeloma cells (**Figure 1A**). Based on these findings, the effects of IL-6 on MM cells were studied. Luciferase-expressing cell lines were subsequently used to assess cell viability. As expected, IL-6 treatment for 48 hours increased the proliferation of RPMI 8226 cells by 1.40-fold (p< 0.0001, **Figure 1B**).

IL-6 can be produced by monocyte-macrophages, which are closely associated with clonal plasma cells in the bone marrow of patients with MM (9). Therefore, we examined whether THP-1 monocytes co-cultured with MM cells had an influence on their proliferation. There was no significant change in the RPMI 8226 cell population after 48 hours of co-culture with increasing numbers of THP-1 cells (**Figure 1B**). In contrast, IL-6 treatment significantly increased RPMI 8226 cell numbers, independent of the presence or absence of THP-1 cells (**Figure 1B**). Subsequently, the effect of IL-6 on THP-1 differentiation was studied. After IL-6 treatment, RNA was extracted from THP-1 cells, and qRT-PCR was performed to quantify the expression of the M1 macrophage-like marker iNOS (**Supplementary Figure S1C**) and the M2 macrophage-like marker CD163 (**Supplementary Figure S1D**). Treatment with 5 ng/mL IL-6 significantly increased the ratio of CD163 to iNOS expression in THP-1 cells compared with the control group without IL-6 treatment (8.61-fold, p < 0.0001, **Figure 1C**). However, a dose-dependent response of IL-6 to THP-1 cell differentiation was not observed in the present study.

### 3.2 M1-like macrophages inhibited RPMI 8226 cell growth with increased apoptosis

**Figure 2:**
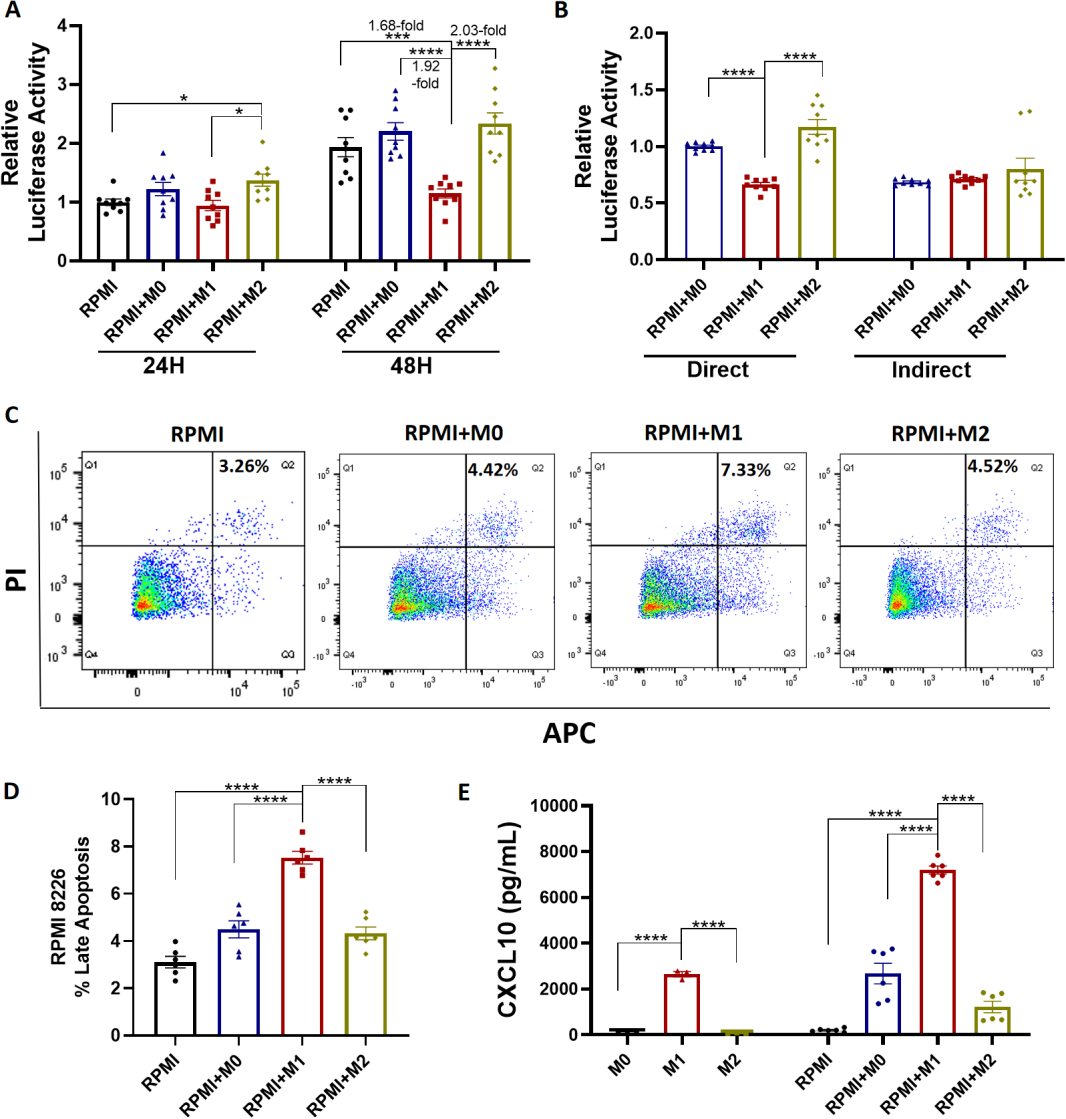
Impact of macrophages on RPMI 8226 cells. **(A)** Luciferase reporter assay of RPMI 8226 cells with or without macrophages for 24, 48 hours at a 1:1 ratio. **(B)** Luciferase reporter assay of RPMI 8226 in the presence of macrophages directly (6-well plate) and indirectly (6-transwell plate) for 48 hours. **(C)** Representative flow cytometry images depicting the RPMI 8226 cells apoptosis by staining with Annexin-V/PI. **(D)** Quantitative analysis of C. **(E)** CXCL10 levels in cell culture supernatants were measured from THP-1 monocyte-derived macrophages and from co-cultures of RPMI 8226 cells with macrophages for 48 hours. M0, THP-1 monocyte-derived M0 macrophages. M1, THP-1 monocyte-derived M1 macrophages. M2, THP-1 monocyte-derived M2 macrophages. RPMI, RPMI 8226 cells. N = 6-9. *p<0.05, **p <0.01, ***p <0.001, ****p <0.0001.

Given the observed increase in M2 macrophage marker CD163 and RPMI 8226 cell numbers following IL-6 treatment, we next investigated the role of macrophages in regulating cell populations by co-culturing RPMI 8226 cells with THP-1 monocyte-derived macrophages. THP-1 monocyte-derived M1 and M2 macrophages were verified by detecting the M1 marker iNOS, M2 marker CD163 and cytokine production (**Supplementary Figures S1E–H**). The proliferation of RPMI 8226 cells was significantly decreased in the presence of M1 macrophages compared to RPMI 8226 alone (1.68-fold, p < 0.01), M0 (1.92-fold, p < 0.0001), and M2 macrophages (2.03-fold, p < 0.0001) after 48 hours at 1:1 ratio (**Figure 2A**). Similar results were observed when RPMI 8226 cells were co-cultured with macrophages at ratios of 2:1 and 1:2 (**Supplementary Figure S4A**). PBMCs-derived macrophages were also used. After 24 hours of co-culture, PBMCs-derived M1 macrophages significantly inhibited the growth of RPMI 8226 cells compared with M0 and M2 macrophages (**Supplementary Figure S3C**). These results were reproduced in other MM cell lines, U266 and MM1.S cells, co-cultured with macrophages for 48 hours (**Supplementary Figures S4B, C**). Furthermore, a 6-well plate transwell system was utilized to investigate whether direct contact between macrophages and RPMI 8226 cells was required to suppress their proliferation. The inhibition of the RPMI 8226 cell population by M1 macrophages was not observed in the transwell system, where macrophages were seeded in the upper chamber and RPMI 8226 cells were seeded in the lower chamber for 48 hours (**Figure 2B**).

In order to study the effects of M1 and M2 macrophages on cancer cells, MM cell (RPMI 8226) apoptosis was measured by flow cytometry after direct co-culture with macrophages. When RPMI 8226 cells were incubated with M1 macrophages, cancer cell apoptotic activity was increased in comparison to the control group (7.33% vs 3.26%, p < 0.0001), as well as cells exposed to M0 (7.33% vs 4.42%, p < 0.0001) and M2 (7.33% vs 4.52%, p < 0.0001) macrophages (**Figures 2C, D**). Similar results were observed in U266 and MM1.S cells (**Supplementary Figure S4D**). Inflammatory cytokines and chemokines play a key role in regulating the progression and development of cancer. CXCL10 is a critical chemokine involved in the recruitment of tumor antigen-specific T cells to the tumor microenvironment. The level of CXCL10 in the cell culture supernatant was significantly increased when M1 macrophages were co-cultured with RPMI 8226 cells for 48 hours (7196 pg/ml vs 205.3 pg/ml, p < 0.0001, **Figure 2E**). However, no significant differences in IL-6 or IL-10 levels were observed in the supernatant of RPMI 8226 cells co-cultured with different types of macrophages (**Supplementary Figures S1G, H**). Overall, the present study suggested that M1 macrophages had a cytotoxic effect on MM cells by promoting apoptosis and increasing CXCL10 release.

### 3.3 Metformin decreased the viability of RPMI 8226 cells

**Figure 3:**
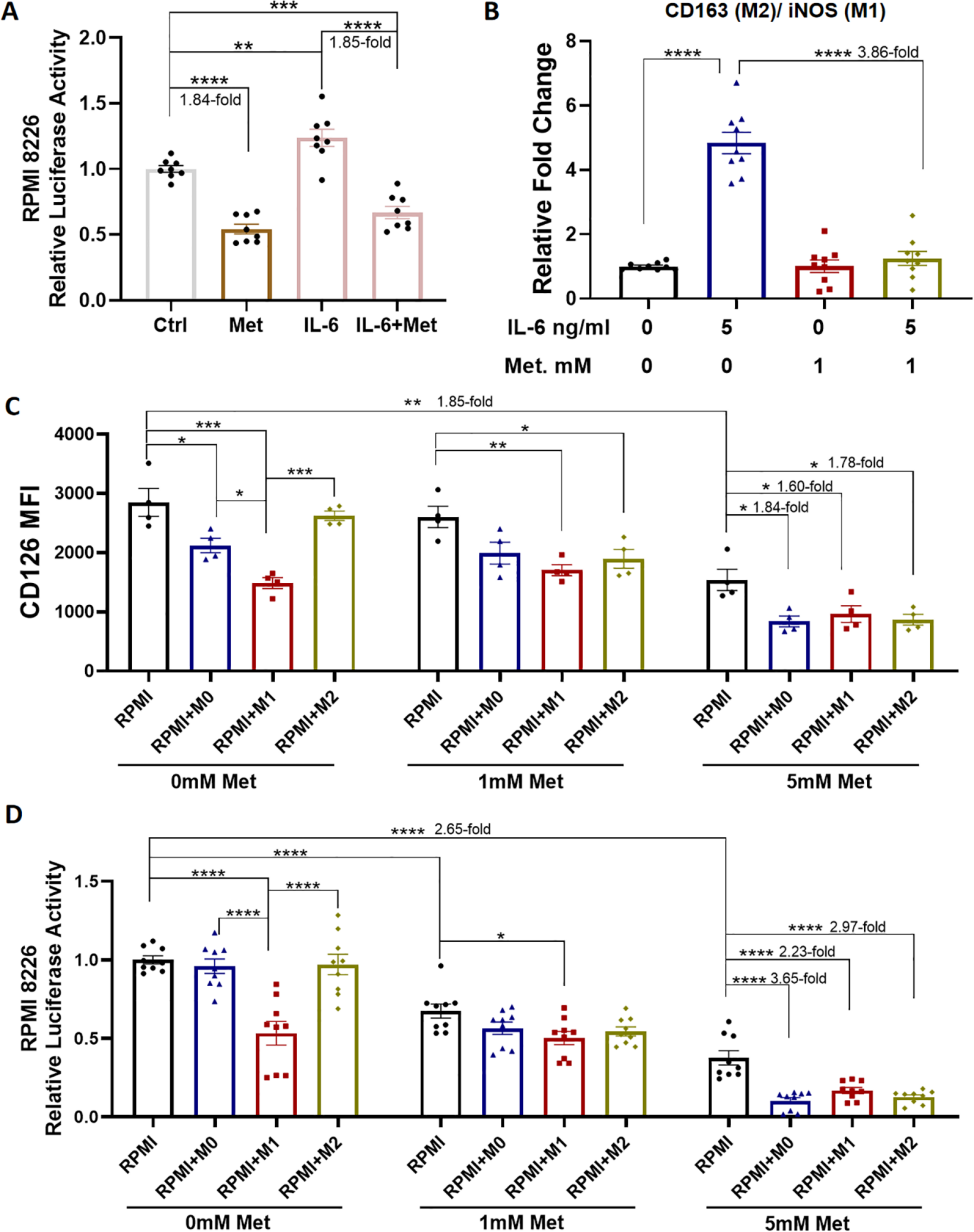
Effects of metformin on RPMI 8226 cells with macrophages. **(A)** Luciferase reporter assay of RPMI 8226 cells following PBS control (Ctrl), metformin (Met. 1mM) or IL-6 (5ng/ml) treatment for 72 hours. **(B)** QRT-PCR analysis of CD163 and iNOS expression in THP-1 cells under various treatment conditions for 72 hours. **(C)** Flow cytometry analysis of CD126 expression on RPMI 8226 cells following 72 hours of metformin treatment with or without macrophages. **(D)** Luciferase reporter assay of RPMI 8226 cells under various treatment conditions at 72 hours. N = 4-9. *p<0.05, **p <0.01, ***p <0.001, ****p <0.0001.

Metformin has been reported to inhibit cancer cell growth by regulating the IL-6 signaling pathway (19). In accordance with previously reported findings, metformin was found to inhibit the proliferation of RPMI 8226 cells, and it effectively suppressed the IL-6–mediated proliferation of these cells (**Figure 3A**). Furthermore, metformin significantly reduced the ratio of CD163 (M2) to iNOS (M1) mRNA expression in THP-1 cells (3.86-fold, p < 0.0001), which had been elevated following 5 ng/ml IL-6 treatment (**Figure 3B**). Flow cytometry was used to examine the effects of metformin on CD126 expression in RPMI 8226 cells in the presence of various types of macrophages. As depicted in **Figure 3C**, CD126 expression was lowest when RPMI 8226 cells were co-cultured with M1 macrophages for 72 hours. Treatment with 5 mM metformin significantly decreased CD126 expression on RPMI 8226 cells (1.85-fold, p < 0.001), and this reduction was even more pronounced in the presence of macrophages (M0: 1.84-fold, M1: 1.60-fold, and M2: 1.78-fold, vs RPMI with 5 mM metformin, p < 0.05, **Figure 3C**). This reduction in CD126 expression by 5 mM metformin was associated with a marked decrease in RPMI 8226 cell viability when co-cultured with M0 macrophages (3.65-fold, p < 0.0001), M1 macrophages (2.23-fold, p < 0.0001) and M2 macrophages (2.97-fold, p < 0.0001), compared to the control group without macrophages (**Figure 3D**). Taken together, these results indicated that metformin suppressed the proliferation of RPMI 8226 cells by down-regulating CD126 expression, and co-culture with macrophages further enhanced this effect.

### 3.4 Macrophages reduced CD126 CAR-T cytotoxicity against RPMI 8226 cells

**Figure 4:**
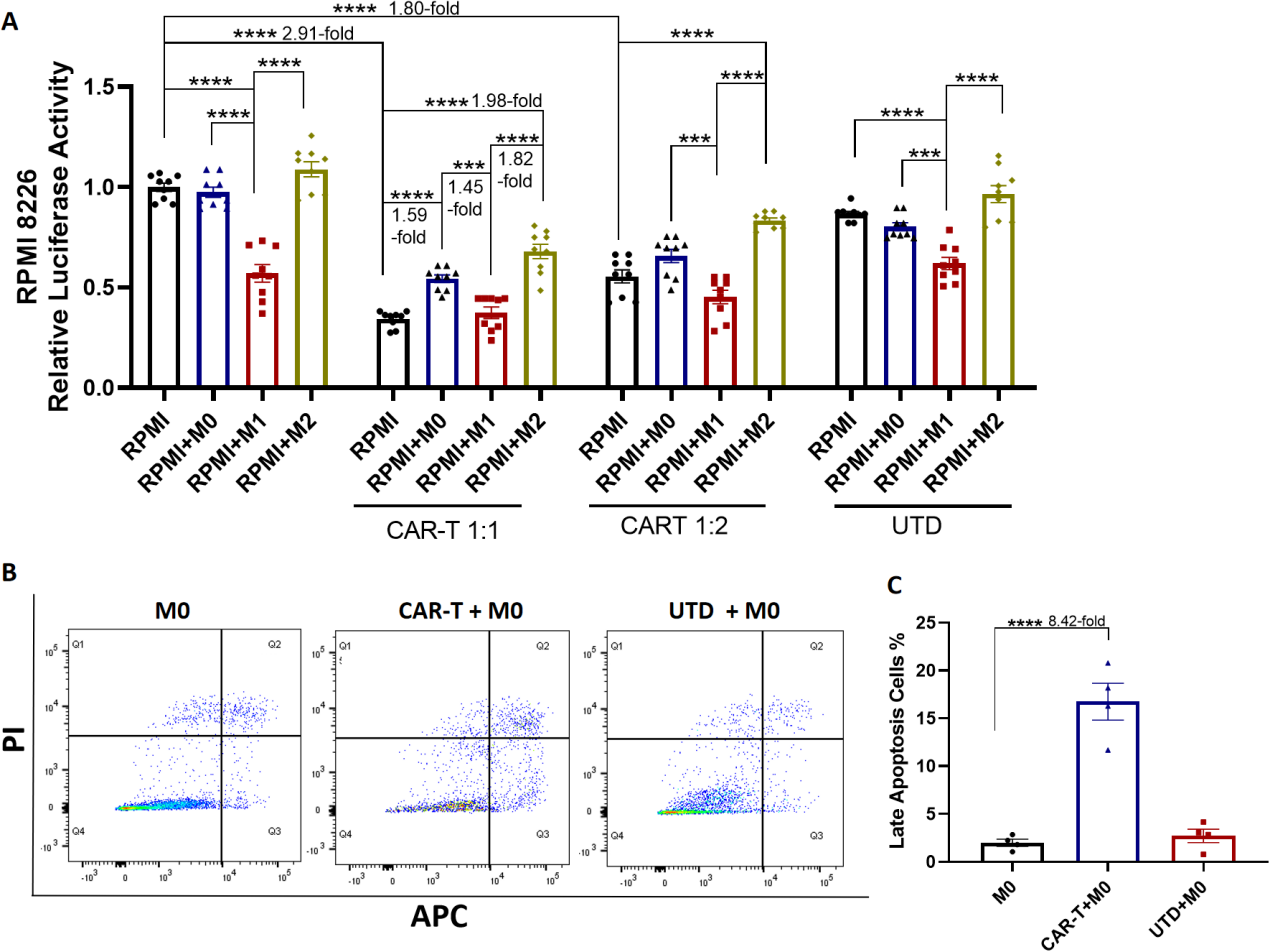
Killing effect of CD126 CAR-T cells on RPMI 8226 cells. **(A)** Viability of RPMI 8226 cells was measured by luciferase assay after 24-hour co-culture with M0, M1, or M2 macrophages, following treatment with either CD126 CAR-T cells or UTD cells at E:T ratios of 1:1 or 1:2 for 24 hours. **(B)** Representative flow cytometry images showing apoptosis of M0 macrophages after 24-hour incubation with CD126 CAR-T or UTD-T cells at a 1:1 ratio, assessed by Annexin V/PI staining of the CD3 negative population. **(C)** Quantitative analysis of B. N = 4-9. *p<0.05, **p <0.01, ***p <0.001, ****p <0.0001.

To further investigate the role of CD126 in regulating RPMI 8226 cell proliferation, CAR-T cells that target CD126 were used. Mock un-transduced T cells were used as a negative control. With CD126 CAR-T cell treatment, the number of RPMI 8226 cells was significantly reduced compared with the untreated control (1:1 ratio of CAR-T cells to RPMI 8226 cells: 2.91-fold decrease, 1:2 ratio: 1.80-fold decrease, p < 0.0001), in a dose-dependent manner (**Figure 4A**). In addition, differentiated macrophages were directly co-cultured with RPMI 8226 cells for 24 hours, followed by an additional 24-hour incubation period with or without CD126 CAR-T cells, to evaluate the impact of macrophages on CAR-T cell activity. As shown in **Figure 4A**, the killing efficacy of CD126 CAR-T cells against RPMI 8226 cells (1:1 ratio) was decreased in the presence of M0 macrophages (1.59-fold vs RPMI, p < 0.0001, 1.45-fold vs RPMI+M1, p < 0.001) and M2 macrophages (1.98-fold vs RPMI, p < 0.0001, 1.82-fold vs RPMI+M1, p < 0.0001). Similar trends were observed with PBMCs-derived macrophages, although the differences were not statistically significant, likely due to the small sample size (**Supplementary Figure S3D**). To further examine this finding, we assessed the killing ability of CD126 CAR-T cells on macrophages. Consistent with previously published studies, CD126 expression was observed in THP-1 cells, as well as in M0, M1 and M2 macrophages (**Supplementary Figure S2A**). Following 24 hours of co-culture, CD126 CAR-T cells significantly increased M0 macrophages apoptosis (8.42-fold, p < 0.0001, **Figures 4B, C**), as well as M1 (3.42-fold, p < 0.05) and M2 macrophages apoptosis (5.57-fold, p < 0.05, **Supplementary Figures S5B–D**), compared with the controls. These findings suggest that macrophages modulated the cytotoxic response of RPMI 8226 cells to CD126 CAR-T cells through their interactions with each other.

### 3.5 Metformin increased CD126 CAR-T cytotoxicity against RPMI 8226 cells

**Figure 5:**
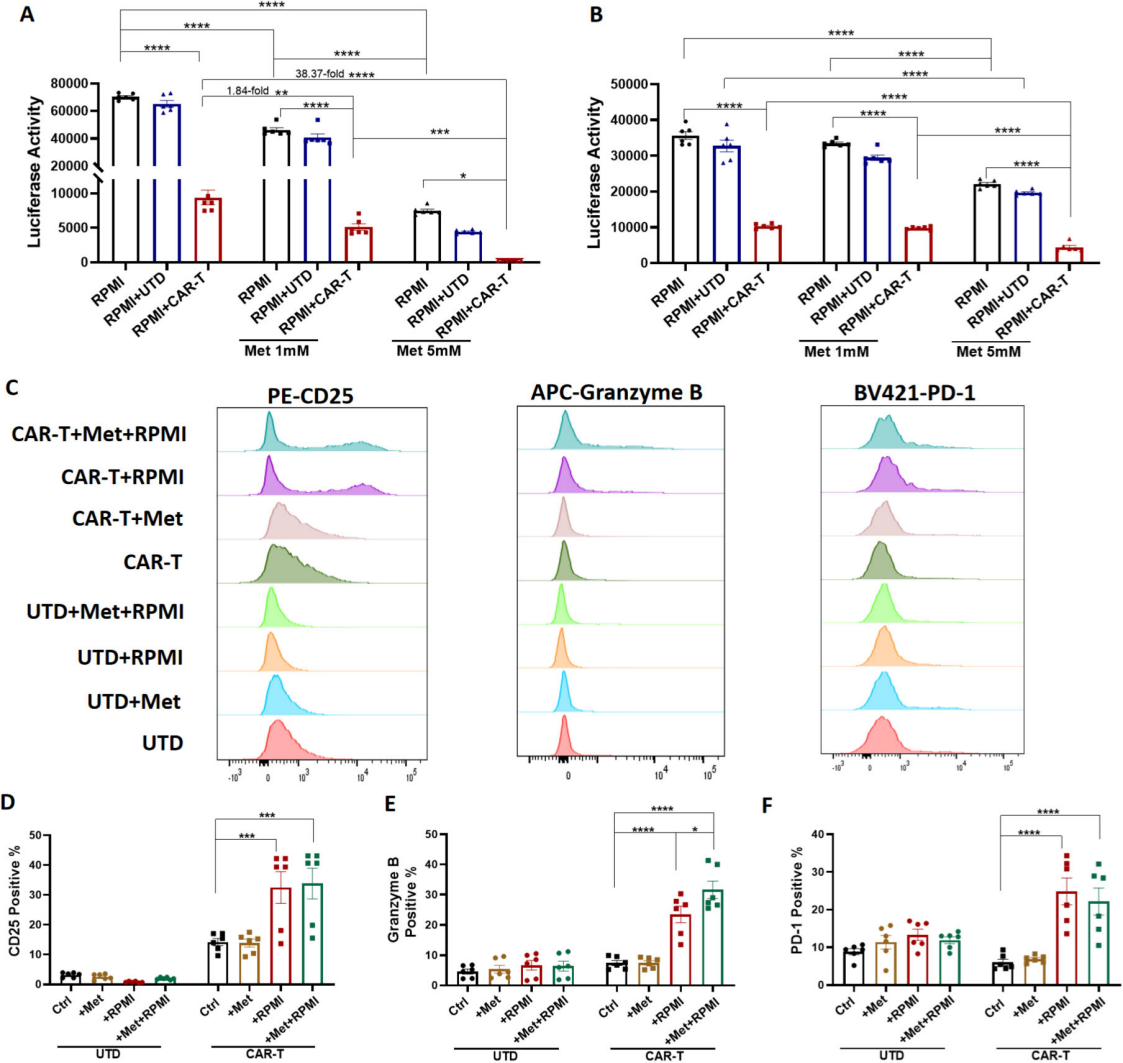
Effects of metformin on CD126 CAR-T cells. **(A)** Luciferase activity of RPMI 8226 cells pre-treated with metformin (1 or 5 mM) for 24 hours, then incubated with CAR-T or UTD T cells at an E:T ratio of 1:1 for an additional 24 hours. **(B)** Luciferase activity of RPMI 8226 cells with treatment of metformin (1 or 5 mM) and CAR-T cells simultaneously at an E:T ratio of 1:1. **(C)** Flow cytometry analysis of CD25, Granzyme B, and PD-1 on T cells after 24-hour metformin (5 mM) treatment or co-culture with RPMI 8226 cells. **(D–F)** Summary data from **(C)** N = 6. *p<0.05, **p <0.01, ***p <0.001, ****p <0.0001.

To determine the potential modulatory effects of metformin on CD126 CAR-T cells, the cytotoxic activity of CD126 CAR-T cells against RPMI 8226 cells, and T-cell function, were evaluated under metformin treatment. RPMI 8226 cells were pre-treated with 1 or 5 mM metformin for 24 hours in a 96-well plate. CD126 CAR-T or UTD T cells were then added at a 1:1 ratio and co-cultured for an additional 24 hours. Remarkably, metformin treatment enhanced the cytotoxic activity of CD126 CAR-T cells against RPMI 8226 cell. As shown in **Figure 5A**, the number of RPMI 8226 cells, as reflected by luciferase activity, further decreased by 1.84-fold and 38.37-fold when co-cultured with CAR-T cells in the presence of 1 and 5 mM metformin, respectively. Similar results were observed when RPMI 8226 cells were treated with metformin and CAR-T cells simultaneously at E:T ratios of 1:1 (**Figure 5B**) and 1:2 (**Supplementary Figure S4E**).

Flow cytometry was used to detect T cell function. The activation marker (CD25), the cytotoxicity marker (Granzyme B) and immune checkpoint receptor (PD-1) were significantly increased in CAR-T cells after co-culture with RPMI 8226 cells for 24 hours, regardless of metformin treatment (**Figures 5C–F**). Metformin treatment alone did not significantly alter CAR-T cell function. However, Granzyme B expression increased when metformin was added to CAR-T cell-treated RPMI 8226 cells (**Figure 5E**). These results suggest that CAR-T cells display a partially activated and potentially exhausted phenotype upon encountering RPMI 8226 cells. Moreover, metformin enhances CAR-T cell-mediated apoptosis of RPMI 8226 cells with increased Granzyme B activity.

### 3.6 CD126 CAR-T cells exhibited cytotoxicity against CD126-negative cells in the presence of macrophages

**Figure 6:**
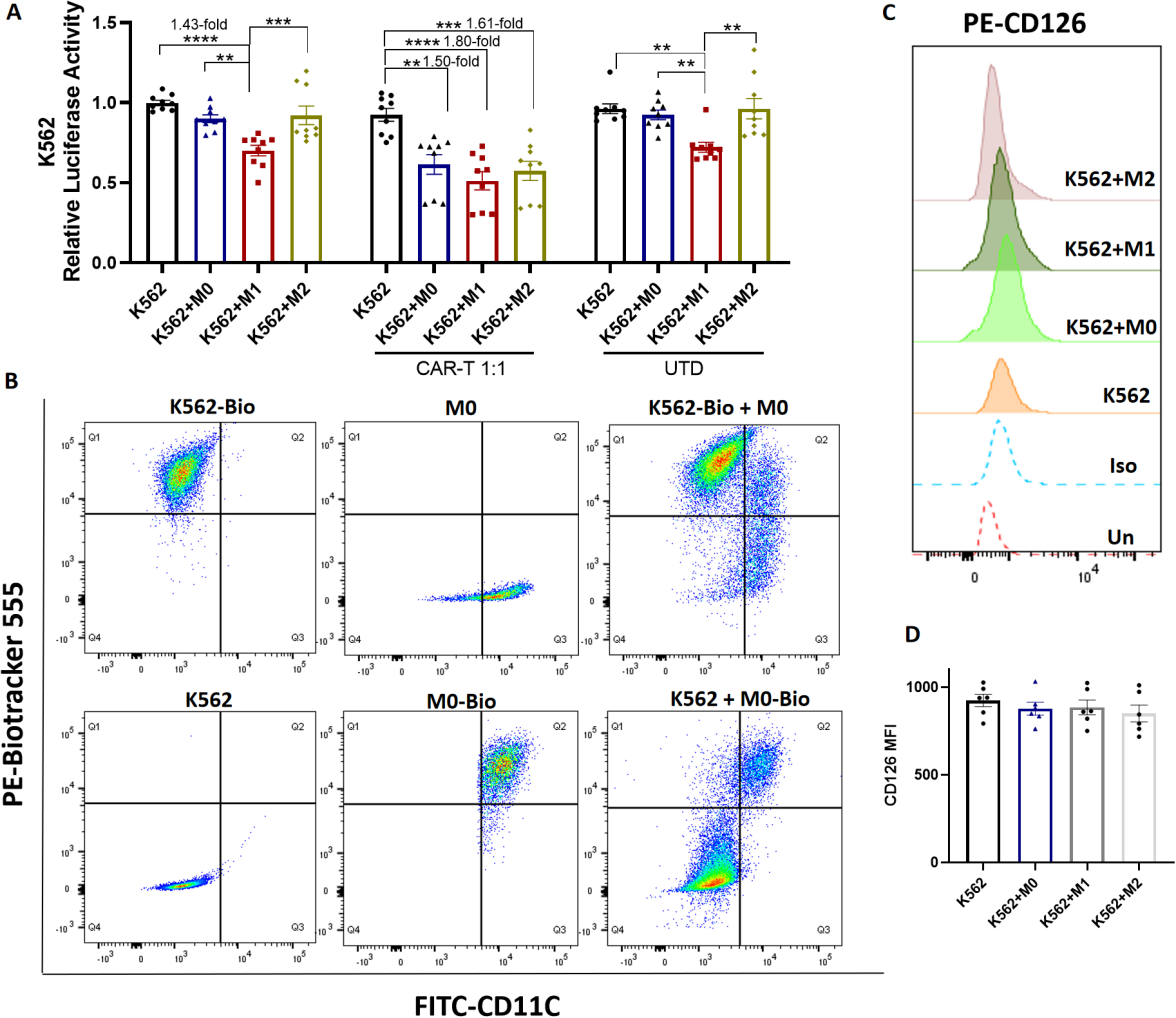
Killing effect of CD126 CAR-T cells on K562 cells. **(A)** Luciferase reporter assay of K562 cells with or without macrophages for 24 hours, following 24-hour co-culture of CD126 CAR-T at an E:T ratio of 1:1. UTD T cells were used as a control. **(B)** Biotracker 555 expression in M0 macrophages (upper panel) or K562 cells (lower panel) was measured by flow cytometry after separate co-culture with Biotracker 555-stained K562 cells or M0 macrophages. CD11C positive cells were M0 macrophages. **(C)** CD126 expression on K562 cells was measured by flow cytometry after co-culture with M0, M1 or M2 macrophages. **(D)** Summary data from **(C)** N = 6-9. *p<0.05, **p <0.01, ***p <0.001, ****p <0.0001.

K562 is a human leukemia cell line that lacks the expression of CD126, serving as a negative control (**Supplementary Figure S2A**). The proliferation of K562 cells was assessed in the presence of M0, M1 and M2 macrophages. Similar to RPMI 8226 cells, M1 macrophages suppressed the proliferation of K562 cells (1.43-fold decrease, p < 0.0001, **Figure 6A**). Next, CD126 CAR-T cells were co-cultured with K562 at 1:1 ratio for 24 hours to detect their killing effect. As expected, CD126 CAR-T cells exhibited no cytotoxic effect on K562 cells (**Figure 6A**), as these cells do not express CD126. Interestingly, a significant reduction of K562 cells following CD126 CAR-T treatment was observed in the presence of M0 (1.50-fold, p < 0.01), M1 (1.80-fold, p < 0.0001) and M2 macrophages (1.61-fold, p < 0.001), compared to conditions without macrophages (**Figure 6A**).

To investigate if the decreased population of K562 cells after CD126 CAR-T treatment was because of the interaction between macrophages and K562 cells, Biotracker 555, a membrane dye, was used to stain cells. As shown in **Figure 6B**, the Biotracker 555-stained K562 cells were CD11C negative, and the CD11C^+^ macrophages did not express any Biotracker 555 signal. Following the co-culture of Biotracker 555-stained K562 cells with M0 macrophages, an observable positive Biotracker 555 signal was detected in the CD11C^+^ M0 macrophage (**Figure 6B**). Similar results were also observed in both M1 and M2 macrophages following co-culture with Biotracker 555-stained K562-GFP cells (**Supplementary Figure S6A**). In contrast, K562 cells did not acquire the BioTracker 555 signal from M0 macrophages after co-culture (**Figure 6B**, lower panel). There was no significant change of CD126 expression on K562 after co-culture with M0, M1 or M2 macrophages compared with K562 cells alone (**Figures 6C, D**). All together, these data indicated that macrophages could interact with K562 cells during the co-culture, and subsequently be targeted and eliminated by CD126 CAR-T cells.

CFSE, which binds to intracellular proteins, can be used to label tumor cells and monitor their phagocytosis by macrophages (33). As shown in **Supplementary Figure S6B**, after 4 hours of co-culture with CFSE-labeled K562 cells, the CFSE signal was significantly increased in M1 macrophages, indicating enhanced phagocytosis. Cytochalasin D, a phagocytosis inhibitor, effectively inhibited macrophage phagocytosis. These results suggest that macrophage-mediated phagocytosis plays a role in the killing of CD126-negative K562 cells.

## 4 Discussion

The dynamic interaction between macrophages and MM cells within the TME plays an important role in disease progression and the response to treatment. TAMs, particularly those polarized to the M2 phenotype, create a pro-tumoral niche that supports tumor cell survival (8). In particular, immunosuppressive macrophages can suppress T-cell activation and induce T-cell exhaustion, which limits the effectiveness of immunotherapies, including CAR-T therapy (14). Additionally, these macrophages contribute to drug resistance by interacting with tumor cells and modulating the bone marrow microenvironment in a way that protects malignant cells from chemotherapy and immunotherapy. This immunosuppressive environment poses significant challenges for achieving durable responses with CAR-T therapy in MM patients. In the present study, we demonstrated that IL-6 increased CD163 mRNA expression in THP-1 cells and promoted RPMI 8226 cells proliferation. In the presence of M1 macrophages, there was decreased cell population and increased apoptosis of RPMI 8226 cells. Metformin reduced the viability of RPMI 8226 cells by reducing CD126 expression in the presence and absence of macrophages. CD126-targeted CAR-T cells significantly reduced RPMI 8226 cell viability, and this effect was further enhanced by metformin treatment. However, the killing efficacy of CD126 CAR-T cells against RPMI 8226 cells was decreased when co-cultured with macrophages. This reduction is likely attributable to the interaction between CD126 CAR-T cells and macrophages. Taken together, these data suggested that macrophages play a critical role in regulating MM cell proliferation and apoptosis, as well as modulating the therapeutic efficacy of metformin and CAR-T cell therapy.

Macrophages are key regulators of inflammatory cytokine production, coordinating immune responses and maintaining tissue homeostasis. It is widely acknowledged that most macrophages originate from monocytes circulating in the peripheral blood (37). M1 phenotype macrophages, activated by pro-inflammatory stimuli such as LPS and IFN-γ, exhibit antigen-presenting capability and produce elevated levels of pro-inflammatory cytokines and chemokines, which can recruit activated T cells, NK cells, and neutrophils to fight infection and tumor cells (38). On the other hand, M2-like macrophages, induced by anti-inflammatory stimuli, such as colony stimulating factor-1 (CSF-1), IL-4, IL-13, and IL-10, are capable of recruiting Th2 cells, contributing to angiogenesis, tissue repair and more importantly tumor progression (39, 40). Higher levels of M2 macrophages are correlated with a poor prognosis in cancer patients. A nanodrug targeting M2-like macrophages was designed to induce M2-to-M1 repolarization through the co-delivery of IKKβ siRNA and the STAT6 inhibitor AS1517499 (AS), thereby suppressing tumor growth and metastasis (41). Another study found that M1 macrophage promoted etoposide-induced apoptosis in HepG2 and A549 cancer cells, whereas M2 macrophages played a protective role by reducing apoptosis in cancer cells exposed to the drug (31). The data from the present study also showed a decreased cell population and an increased apoptosis rate of RPMI 8226 cells in the presence of M1 macrophages.

IL-6 and its receptor are important for the growth and survival of multiple types of solid and hematologic malignancies (42). IL-6 signaling has been shown to modulate the tumor immune microenvironment by promoting the polarization of macrophages toward the M2 phenotype, inhibiting cytotoxic T cell responses, and enhancing the activity of Tregs, thereby contributing to an immunosuppressive environment that facilitates tumor progression (18). It is well documented that IL-6 plays a key role in the development of myeloma by promoting the proliferation and preventing apoptosis of malignant plasma cells (43, 44). Indeed, in the present study, we observed that IL-6 promoted RPMI 8226 cells proliferation. IL-6, although predominantly secreted by M1 macrophages, is paradoxically associated with tumor-promoting effects, despite the well-documented anti-tumor function of M1 macrophages. This paradox may be explained by the pleiotropic nature of IL-6, which can exert either pro- or anti-tumor effects depending on the cellular context and microenvironmental conditions (18).

CAR-T cell therapy has improved MM treatment, especially with the B-cell maturation antigen(BCMA)-targeted CAR-T cells (12). However, the long-term efficacy of CAR-T therapy is hindered by several factors, including the immunosuppressive influence of the TME (14). The presence of an immunosuppressive TME limits CAR-T cell efficacy by recruiting immunosuppressive Treg cells, myeloid-derived suppressor cells (MDSCs), and TAMs. An increased frequency of CD14^+^ CD68^+^ TAMs has been observed in MM patients, where they contribute to the suppression of cytotoxic T lymphocyte (CTL) activity via the PD-1/PD-L1 signaling pathway (45). CD8^+^ T cells exhibit prolonged interactions with TAMs, which reduces their migration and infiltration into tumor nests. Following TAM depletion, T cell migration and infiltration into tumor tissue were restored (46). However, the direct interaction between CAR-T cells and macrophages has not been well studied. In this study, we observed that the killing efficacy of CD126 CAR-T cells against RPMI 8226 cells was reduced when co-cultured with macrophages. Future strategies aimed at reprogramming or depleting TAMs may enhance the efficacy of CAR-T cell therapy in patients with MM.

Metformin, has been shown to have immunomodulatory effects, including the ability to shift macrophage polarization from the pro-tumoral M2 phenotype to the anti-tumoral M1 phenotype (47, 48). Treatment with metformin (0.25–2 mM for 48 h) dose-dependently decreased the number of M2 macrophages derived from PMA-induced THP-1 macrophages (49). Metformin was loaded into mannose-modified macrophage-derived microparticles to efficiently target M2 macrophages and promote their repolarization into an M1-like phenotype (50). The clinical effect of metformin on breast cancer patients has been investigated. Lower proportions of CD163^+^ M2-TAMs and higher ratios of CD3^+^ and CD8^+^ T cells were observed in breast cancer patients with pre-existing Type 2 Diabetes Mellitus who received metformin (750–1500 mg/day) (51). In the present study, we also found that metformin reduced the M2 (CD163) to M1 (iNOS) marker ratio in THP-1 cells, favoring a tumor-suppressive phenotype. It should be noted that metformin has also been reported to promote M2 macrophage polarization in some studies (52, 53). Macrophage polarization can vary markedly across different pathological states. The impact of metformin on M1/M2 macrophage polarization is complex and depends on factors such as disease context, dosage, and treatment duration (27). Moreover, metformin has been reported to enhance the memory features of CD8^+^ T cells, contributing to greater anti-tumor efficacy (29). A recent study demonstrated that pretreating CAR-T cells with both metformin and rapamycin activated peroxisome proliferator-activated receptor gamma coactivator 1-alpha (PGC-1α) through mechanistic target of rapamycin (mTOR) inhibition and AMP-activated protein kinase (AMPK) activation, leading to increased mitochondrial spare respiratory capacity and enhancing CAR-T cell resistance to exhaustion (54). This metabolic enhancement resulted in sustained and effective anti-glioma cytotoxic activities under hypoxic conditions, significantly extending survival in glioma-bearing mice (54). By modulating both macrophage polarization and T-cell function, metformin presents a potential therapeutic strategy to overcome TME-induced resistance to CAR-T therapy. Here, we demonstrated that metformin decreased the viability of RPMI 8226 cells, which exhibited reduced CD126 expression, in the presence of macrophages. Recent studies also indicate that combining metformin with CAR-T therapy may enhance both the response and durability of MM treatment (55, 56).

It is important to acknowledge the limitations associated with using THP-1 cells-derived macrophages. THP-1 cells are derived from acute monocytic leukemia and therefore may differ from primary human monocytes/macrophages in proliferation, surface marker expression (e.g., CD14 and CD16), and innate immune signaling responses, including Toll-like receptor-mediated pathways (57, 58). In addition, macrophage differentiation in THP-1 cells typically requires pharmacologic stimulation (e.g., PMA), which may not fully recapitulate physiological monocyte-to-macrophage differentiation (59). Other cell types, such as PBMCs, were needed to verify the results. Macrophages were classified into two types, M1 and M2, by Mills et al. in 2000 (60). As research has continued, many newly identified macrophage phenotypes *in vivo* have been found to lie between these two extremes, such as M2a, M2b, M2c, and M2d macrophages, based on activation stimuli and their function (61). Therefore, although the present findings provide mechanistic insights under controlled experimental conditions, further studies using models that better recapitulate the complexity of the tumor microenvironment are needed to validate their physiological and translational relevance.

The complex interactions among macrophages, MM cells, and CAR-T cells within the TME highlight the need for comprehensive therapeutic strategies. The approaches that can be used to enhance CAR-T cell efficacy include, but are not limited to, metabolic modulation, selective macrophage depletion or reprogramming, optimization of CAR affinity and targeting, and strategic sequencing of interventions, to both preserve beneficial macrophage functions and mitigate negative impacts on CAR-T therapy outcomes. For example, preclinical studies have shown that CAR-T cells targeting suppressive TAM markers, such as TREM2, can deplete TAMs *in vivo*, reprogram the tumor microenvironment, enhance endogenous T-cell infiltration and activation, and induce tumor regression without causing systemic toxicity (62). The findings from the present study provide guidance for future research aimed at overcoming the limitations of current MM treatments and developing more durable, long-lasting therapeutic strategies.

## Data Availability

The original contributions presented in the study are included in the article/Supplementary Material. Further inquiries can be directed to the corresponding author.
