# Supplementary material for: Macrophage and IL-6 signaling modulate multiple myeloma progression and response to metformin and CAR-T cell therapy
Source: Front Immunol. 2026 Apr 16;17:1760136. doi: 10.3389/fimmu.2026.1760136 (PMC13128645; doi:10.3389/fimmu.2026.1760136)
Supplement: Supplementary file 1 [file DataSheet1.pdf]

Supplementary Material

Supplementary Figures

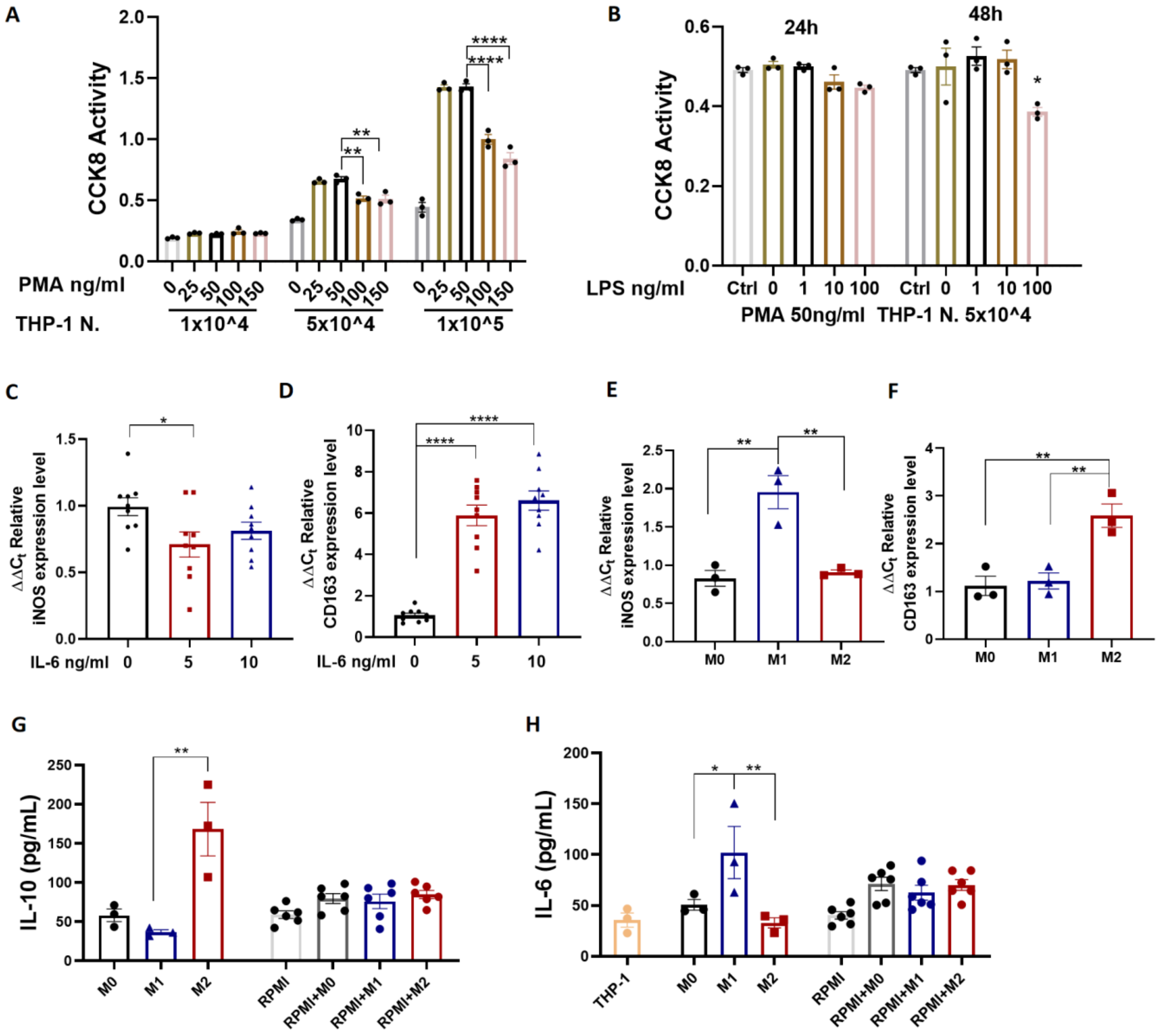

Supplementary Figure S1. Analysis of mRNA expression and cytokine production in THP-1 cells.

(A) CCK-8 assay of M0 macrophages derived from THP-1 cells, incubated for 24 hours with varying concentrations of PMA, followed by an additional 24-hour incubation in RPMI medium in 96-well plates. (B) Effects of LPS on THP-1-derived macrophage viability after 24 or 48 hours of incubation. Relative mRNA expression of iNOS (C) and CD163 (D) in THP-1 cells following IL-6 treatment, measured by qRT-PCR. The mRNA expression of M1 macrophage markers (E) and M2 macrophage markers (F) was studied by qRT-PCR in THP-1-derived macrophages. Levels of the pro-

inflammatory cytokine IL-6 (G) and the anti-inflammatory cytokine IL-10 (H) in cell culture supernatants were quantified by ELISA. Analyses were performed on supernatants from THP-1 monocyte-derived M0, M1 and M2 macrophages, RPMI 8226 cells, and RPMI 8226 cells co-cultured with M0, M1 and M2 macrophages. N=3-6. * $p<0.05$, ** $p<0.01$, *** $p<0.001$, **** $p<0.0001$.

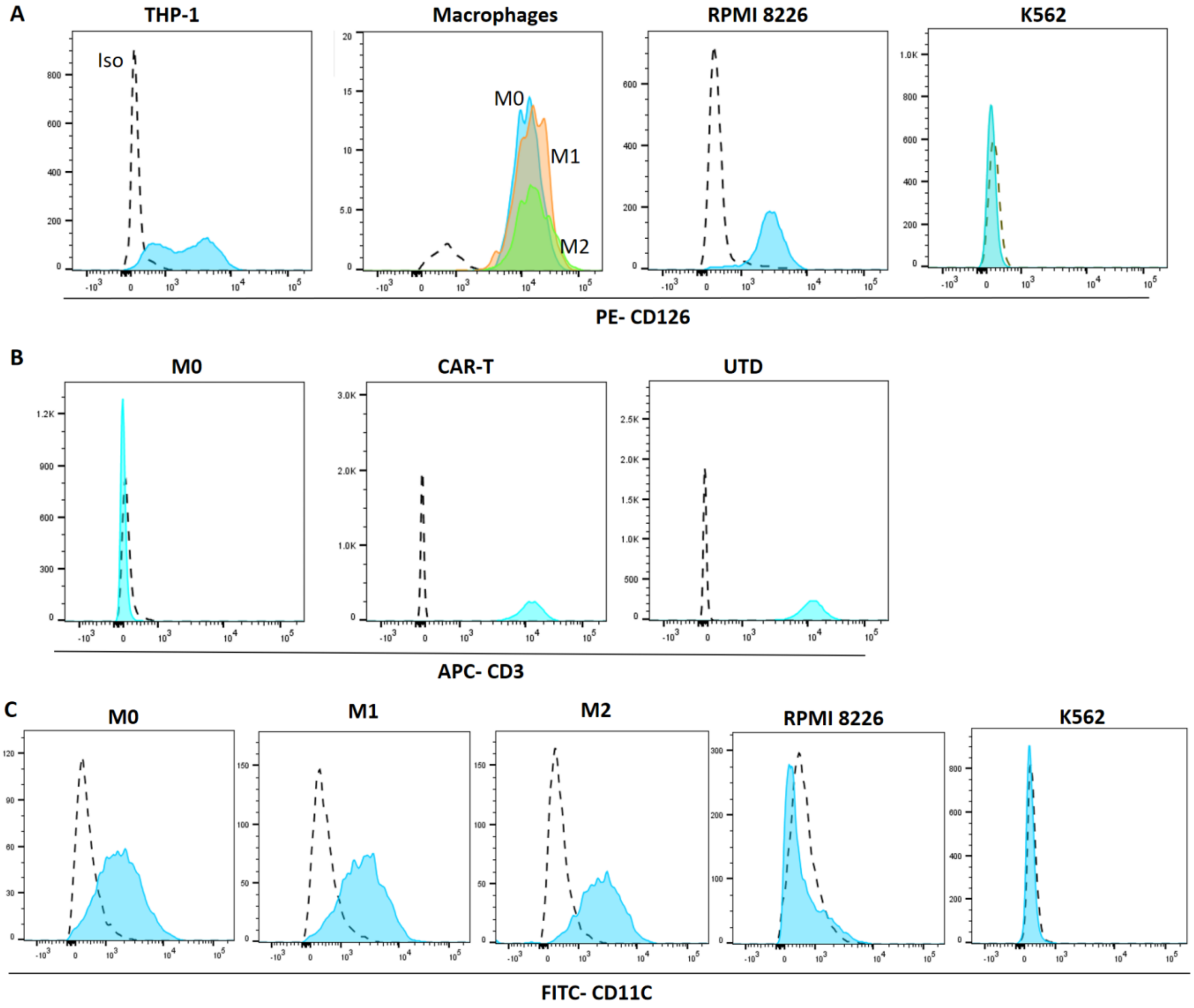

Supplementary Figure S2. Flow cytometry analysis of cell-specific markers.

(A) CD126 expression on THP-1, macrophages, RPMI 8226 and K562 cells. (B) CD3 expression on M0 macrophages, CD126 CAR-T and UTD-T cells. (C) CD11C expression on macrophages, RPMI 8226 and K562 cells. Iso: isotype control.

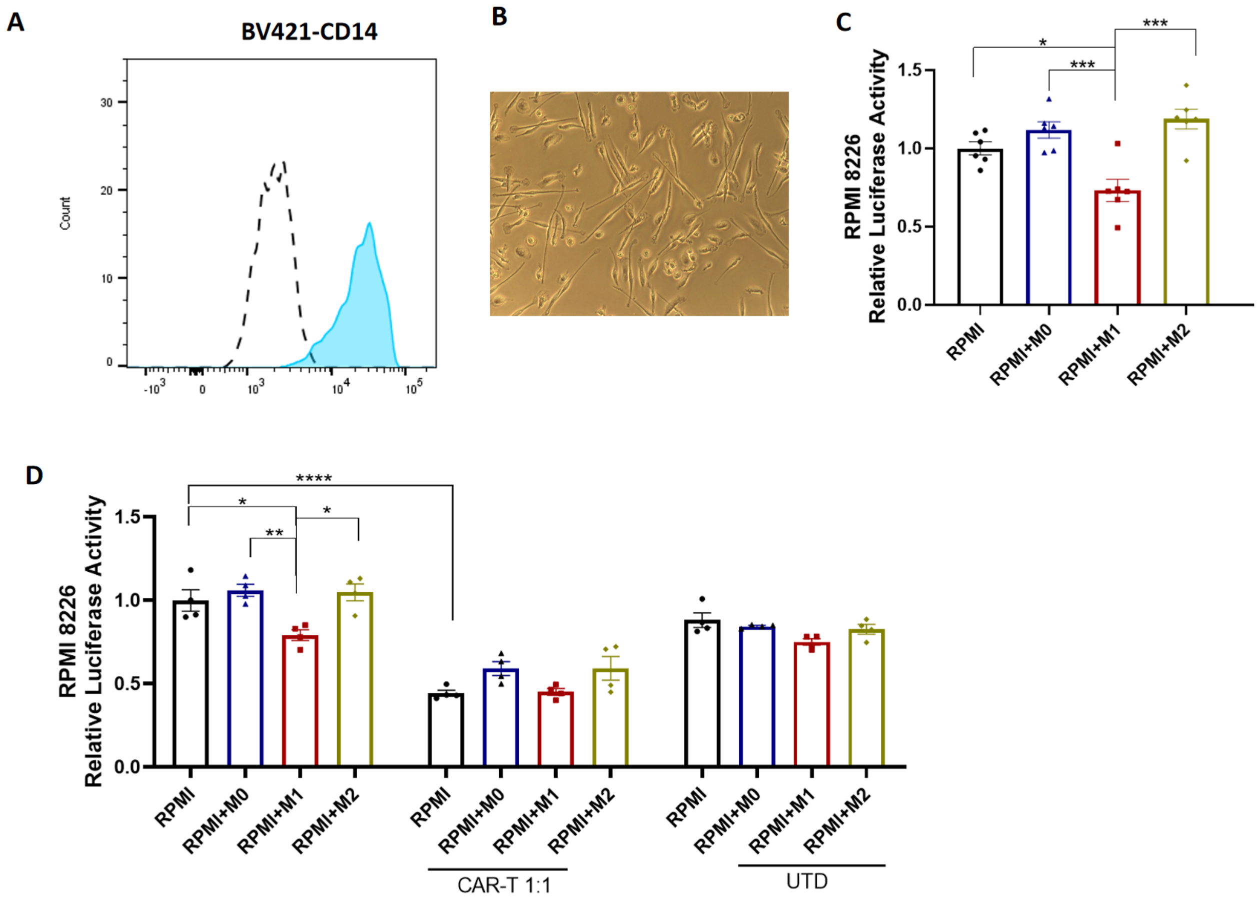

Supplementary Figure S3. Effects of PBMCs-derived macrophages on RPMI 8226 cells.

(A) CD14 expression was detected on PBMC-derived macrophages by flow cytometry. (B) Light microscopy image of PBMC-derived M0 macrophages at 10x objective magnification. (C) Luciferase reporter assay of RPMI 8226 cells co-cultured with or without PBMCs-derived macrophages (M0, M1, or M2) at a 1:1 ratio for 24 hours. (D) Viability of RPMI 8226 cells was determined by luciferase assay after 24-hour co-culture with PBMCs-derived M0, M1, or M2 macrophages, followed by incubation with CD126 CAR-T cells or UTD T-cells at an E:T ratio of 1:1 for 24 hours. N=4-6. * $p<0.05$, ** $p<0.01$, *** $p<0.001$, **** $p<0.0001$.

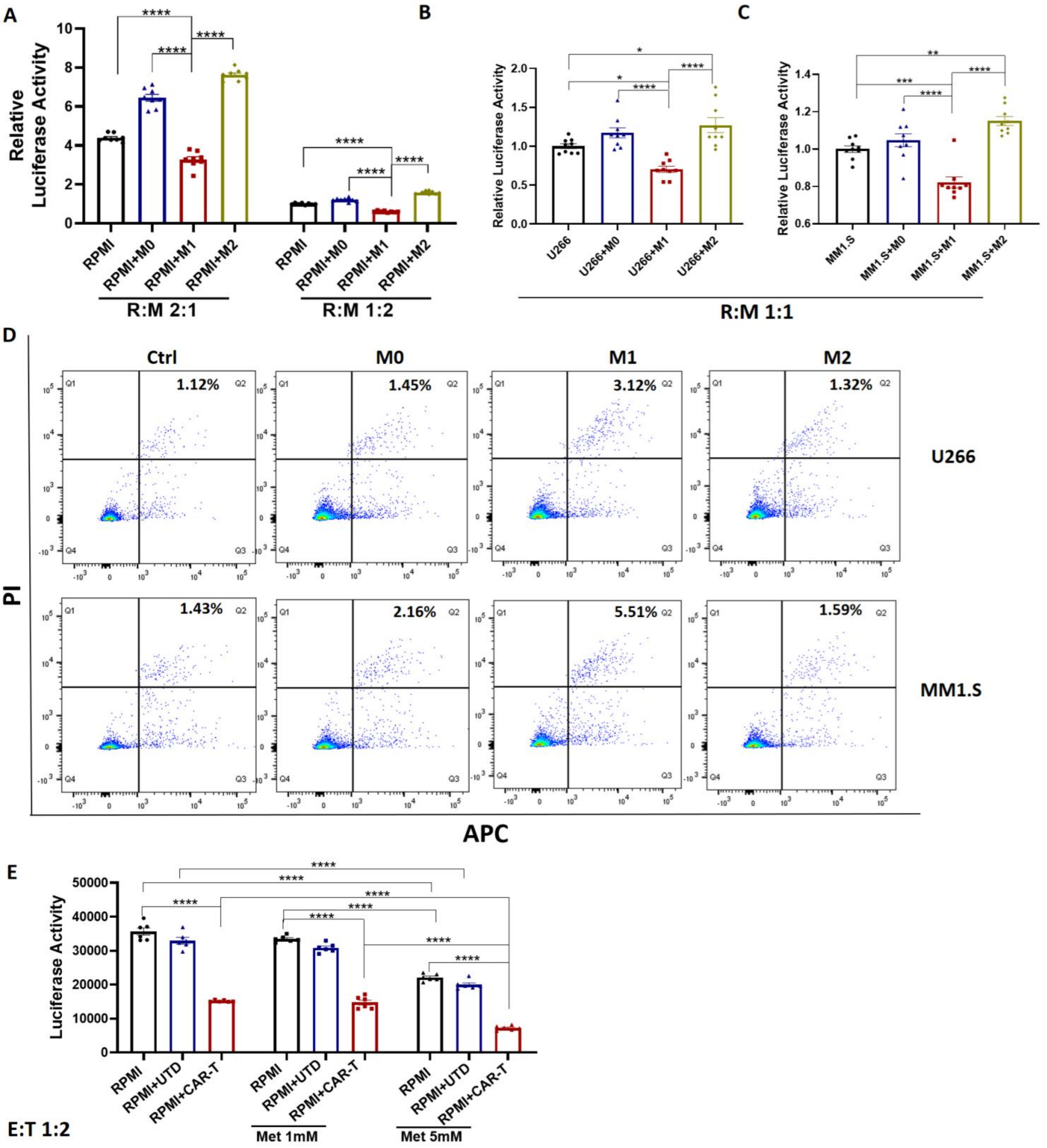

Supplementary Figure S4. Effects of THP-1 monocyte-derived macrophages on MM cells.

Luciferase reporter assay of (A) RPMI 8226 cells, (B) U266 cells, and (C) MM1.S cells with or without macrophages for 48 hours at various ratios. (D) Representative flow cytometry images showing apoptosis of MM cells stained with Annexin V/PI. Ctrl: MM cells alone; M0: MM cells co-cultured with THP-1-derived M0 macrophages; M1: MM cells co-cultured with THP-1-derived M1 macrophages; M2: MM cells co-cultured with THP-1-derived M2 macrophages. (E) Luciferase activity of RPMI 8226 cells with treatment of metformin (1 or 5mM) and CAR-T cells simultaneously at an E:T ratio of 1:2. N=6-8. * $p<0.05$, ** $p <0.01$, *** $p <0.001$, **** $p <0.0001$

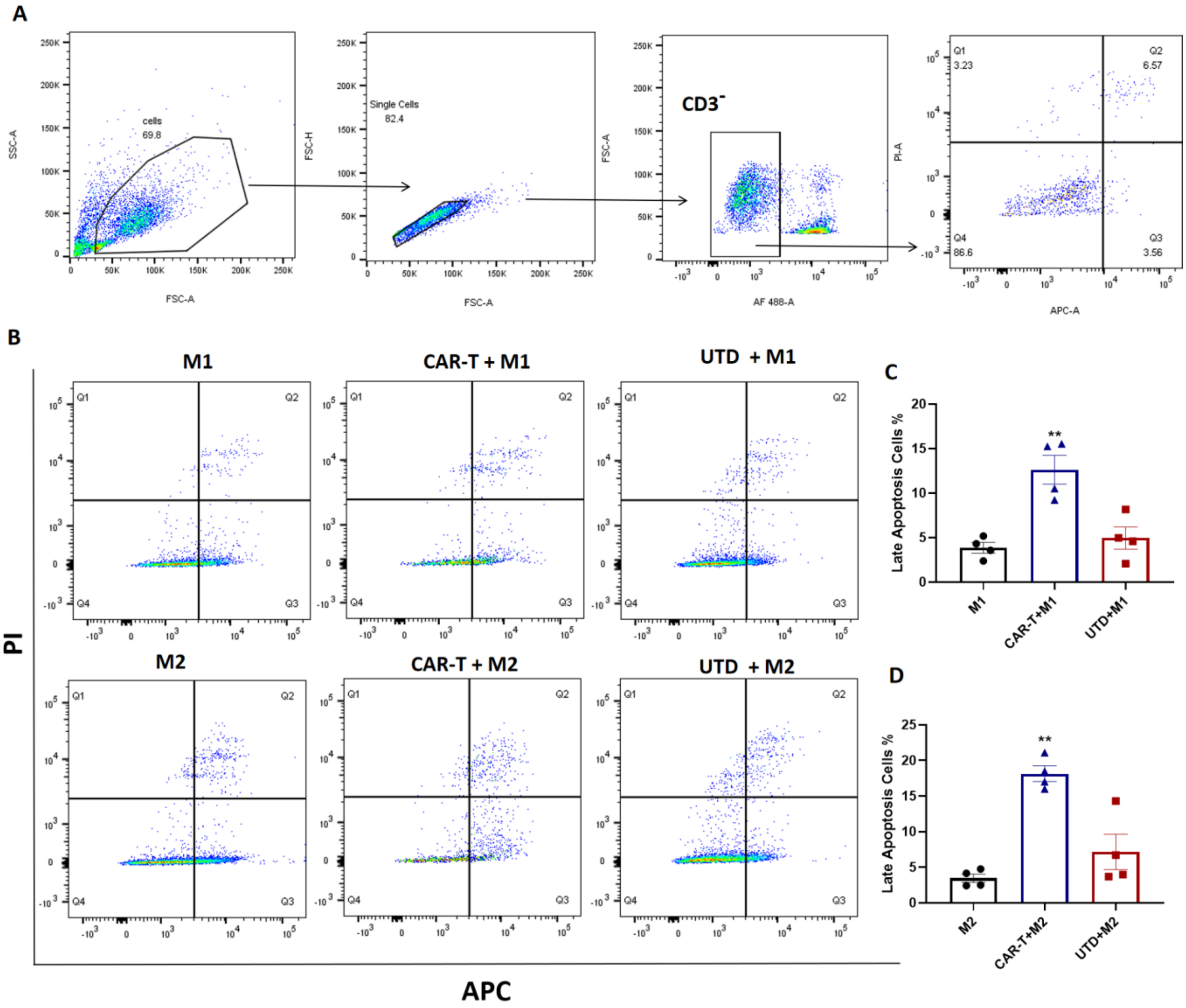

Supplementary Figure S5. CAR-T cell-mediated apoptosis of macrophages.

(A) Flow cytometry gating scheme for the apoptosis of CD3 negative macrophages. (B) Representative flow cytometry images showing the M1 macrophages apoptosis (upper panel) and M2 macrophages apoptosis (lower panel) after incubation with CD126 CAR-T or UTD T cells at an E:T ratio of 1:1 for 24 hours, as assessed by Annexin V/PI staining from CD3- population. Quantitative analysis of M1 macrophages apoptosis (C) and M2 macrophages apoptosis (D). N=4. * $p<0.05$.

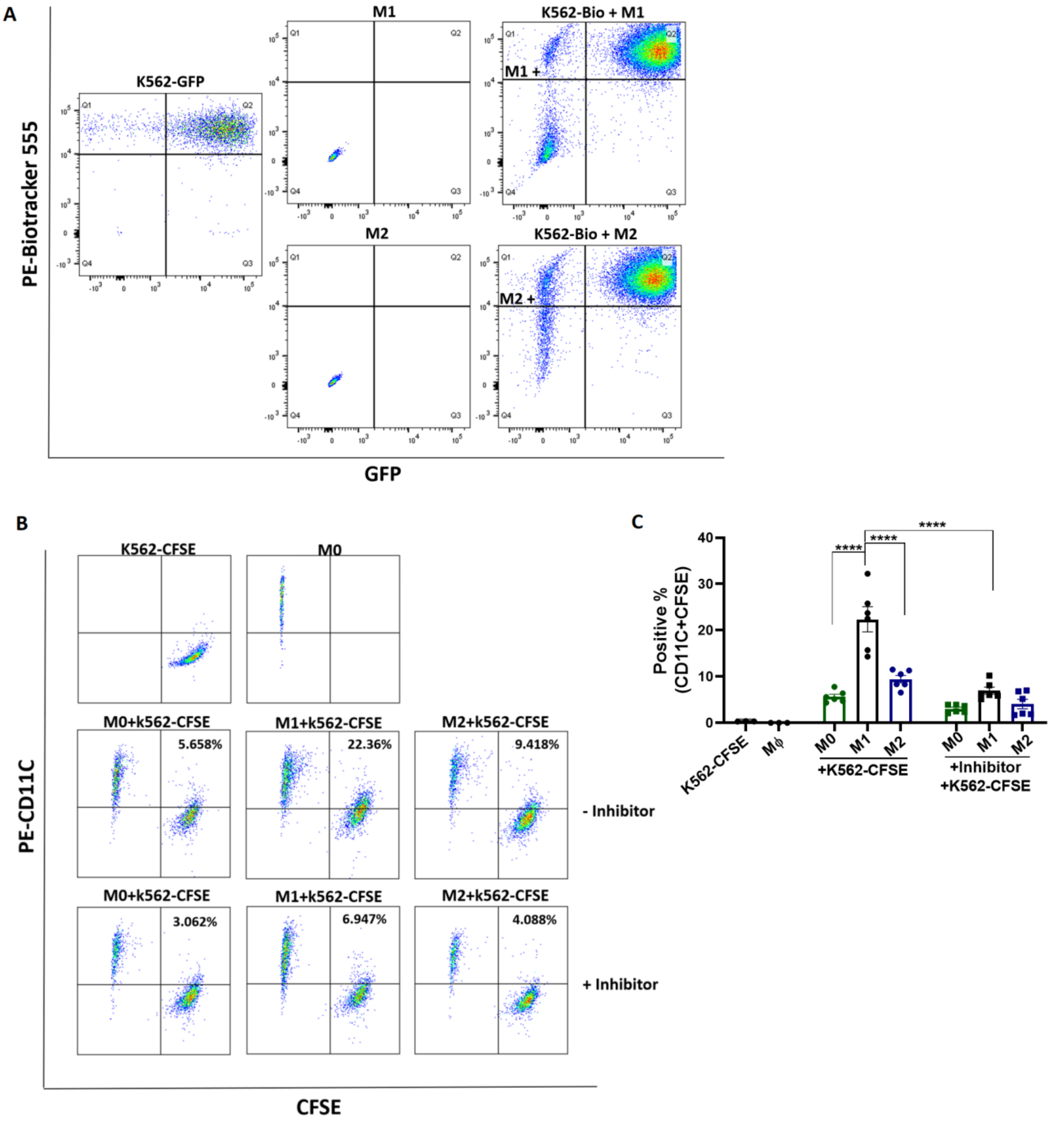

Supplementary Figure S6. Interactions between K562 cells and macrophages.

(A) Flow cytometry analysis of M1 (upper) and M2 (lower) macrophages shows the uptake of BioTracker 555 after co-culture with K562-GFP cells. K562 cells are GFP positive, allowing them to be distinguished from GFP negative macrophages. (B) Flow cytometry was used to detect phagocytosis of CFSE-labeled K562 cells by M0, M1, and M2 macrophages, with or without a phagocytosis inhibitor (Cytochalasin D). The phagocytosis efficiency is represented by the

percentage of $CD11C^{+}/CFSE^{+}$ cells. (C) Quantification of macrophage phagocytosis. N=6. * $p<0.05$, ** $p<0.01$, *** $p<0.001$, **** $p<0.0001$.
